# Reimagining the future of African brain health: Perspectives for basic research on the pathogenesis of cryptococcal meningitis

**DOI:** 10.1016/j.bbih.2021.100388

**Published:** 2021-11-12

**Authors:** R. Dangarembizi

**Affiliations:** a Division of Physiological Sciences, Department of Human Biology, Faculty of Health Sciences, University of Cape Town, Cape Town, South Africa; b Neuroscience Institute, Faculty of Health Sciences, University of Cape Town, Groote Schuur Hospital, Cape Town, South Africa; c CMM AFRICA Medical Mycology Research Unit, Institute of Infectious Disease and Molecular Medicine, Faculty of Health Sciences, University of Cape Town, Cape Town, South Africa

**Keywords:** Neuroinflammation, Cryptococcus, Neuroimmune responses, Signalling pathways

## Abstract

Cryptococcal meningitis is a fatal opportunistic infection of the brain and a leading cause of neurological damage and death in immunocompromised individuals. This neglected fungal disease of the brain is a huge burden on the health systems of developing countries, especially in Sub-Saharan Africa, where up to 25% of people living with HIV/AIDS succumb to it. Cryptococcal fungal cells have a predilection for the brain and they are capable of traversing the blood brain barrier and invade the brain where they cause infection, inflammation and a disruption of normal brain function. A robust host neuroimmune response is critical for pathogen clearance and survival, and a good understanding of the mechanisms underlying its development in the host is critical for the development of effective treatments. However, past basic research studies have been focussed on the characteristics of the fungus and its effect on the peripheral immune system; with little attention paid to how it interacts with brain immune cells. This mini review briefly discusses the paucity of basic research data on the neuroimmune response to cryptococcal infection, raises pertinent questions on how the brain cells respond to the fungal infection, and thereafter discusses models, techniques and advanced technologies that could be useful for carrying out high-throughput research on the pathogenesis of cryptococcal meningitis.

## 1 Cryptococcal meningitis: a neglected threat to African brain health

Cryptococcal meningitis is a highly invasive and deadly fungal infection of the central nervous system (CNS), predominantly prevalent in immunocompromised individuals, although it also affects patients with no history of immunosuppression (Rodrigues, 2018). The burden of cryptococcosis on African health systems is a complex issue that is best discussed in the context of the multiple factors that contribute to it. In 2020, there were 38 million people living with the human immunodeficiency virus and/or acquired immunodeficiency syndrome (HIV/AIDS) globally; more than 80% of whom were from Sub-Saharan Africa (SSA) (Global, 2020). Owing to this high burden of HIV/AIDS, SSA has the highest prevalence of cryptococcal meningitis. In the past 20 years, the annual incidence of cryptococcal meningitis rose to reach a staggering 957 ​900 new cases and 624 ​700 deaths (Park et al., 2009). While the introduction of antiretroviral therapy (ART) has since significantly reduced the prevalence of cryptococcal meningitis in developed countries, developing parts of the world continue to see a high prevalence of the disease. Despite the introduction of ART, SSA still contributes almost 75% of the global share of new cases and deaths (Mandengue and Denning, 2018; Schwartz et al., 2019; Nyazika et al., 2018; Rajasingham et al., 2017), mainly because of lack of adherence to ART, ART failure and a paradoxical manifestation of cryptococcal disease in patients recruited to ART (Jarvis et al., 2009). Mortality rates associated with cryptococcal meningitis are high; with 10-week mortality lying between 20 and 60% with treatment, and up to 100% without treatment (Molloy et al., 2017). In low-to-medium income countries (LMICs), the high mortality rate is due to the unavailability and inaccessibility of safe and effective drugs. The recommended treatment regime includes a two-week induction on a combination of amphotericin B and flucytosine followed by maintenance on fluconazole for up to eight weeks post-hospitalisation (Loyse et al., 2019; Rajasingham et al., 2012; Rothe et al., 2013). However, the cost of these drugs is beyond the affordability of much of the affected population, who live in rural or poor urban areas of LMICs with no access to proper health care facilities. In addition, the lack of facilities and/or capacity to intravenously administer amphotericin B and carry out intensive laboratory testing for its toxicity renders this treatment option inaccessible (Loyse et al., 2019; Rajasingham et al., 2012). As a result, treatment is usually limited to fluconazole monotherapy, which has poor treatment outcomes (Loyse et al., 2019; Rothe et al., 2013; Bicanic et al., 2006; Mwaba et al., 2001). The cost of cryptococcal-related morbidity and mortality on African brain health (and subsequently all of Africa's development) is underreported but certainly not beyond imagination. Although not listed as one, cryptococcal meningitis squarely fits the World Health Organisation (WHO)'s definition of a neglected tropical disease: a tropical disease that disproportionately affects poor and underdeveloped populations and contributes to high morbidity and mortality (Molloy et al., 2017). It is surprising how this disease remains neglected in research, funding and policy, yet it claims as much, or potentially more lives of people living with HIV/AIDS than does tuberculous meningitis (Rodrigues, 2018; Molloy et al., 2017).

## 2 A dearth in basic research studies focussing on neuroimmune responses to cryptococcal infections

Lethal cryptococcosis is observed when the fungus invades the CNS (Terada, 2010) and the host neuroimmune response to the pathogen is a critical determinant of whether one survives or succumbs to infection. In HIV-related neurocryptococcosis, where cell-mediated and humoral immune defences are suppressed, innate neuroimmune function is unquestionably critical for pathogen clearance. Survival and mortality in cryptococcal infections is dependent upon the delicate balance between the pathogen's virulence and the effectiveness of the host immune system (Chayakulkeeree and Perfect, 2008), but current research in cryptococcal infections leans heavily towards fungal biology and peripheral immune responses than it does on host neuroimmune mechanisms. Basic research on cryptococcosis has not been focussed on the development of the disease in the brain. In their analysis of the abstracts presented at the 9th International Conference on Cryptococcus and Cryptococcosis, arguably the biggest scientific meeting on the subject of cryptococcal infections, Colombo and Rodrigues (2015) showed that research focussing on brain pathogenesis contributed a minor fraction of the research activities at the meeting. Similarly, basic research studies focussing specifically on mechanisms of neurocryptococcosis are very hard to come by in refereed literature. In contrast, a myriad of clinical studies have been done which have shaped our knowledge of the epidemiology, fungal characteristics, diagnosis, pathogenesis, and management of cryptococcal meningitis (Bicanic et al., 2007; Bicanic et al., 2009a; Bicanic et al., 2009b; Klock et al., 2008; Molloy et al., 2021; Robertson et al., 2014; Sharma et al., 2010; Vlasova-St Louis et al., 2021a). However, a deeper understanding of basic cryptococcus-brain interactions is still required at the cellular and molecular level to enable identification of molecular targets and the development of safer and more effective treatments. The aim of this review is to probe current knowledge and point out pertinent questions that remain unanswered on the basic neurobiology of cryptococcal infections. The review also aims to briefly discuss experimental models or techniques that could be useful as tools for scaling up basic research data on the neuroimmune response to cryptococcosis of the brain.

## 3 Pathogenesis and clinical manifestations of cryptococcal meningitis

Cryptococcosis is caused by the bacidiomycetes *Cryptococcus neoformans* (serotype A, D and AD) and *Cryptococcus gattii* (serotype B and C) (Maziarz and Perfect, 2016; Velagapudi et al., 2009). *C. neoformans* is ubiquitous in the environment but it is mainly found in contaminated soil, decaying wood and pigeon droppings (Levitz, 1991), while *C. gattii* is found in tropical and subtropical regions and usually associated with eucalyptus trees. *C. neoformans* (serotype A) causes almost 95% of cryptococcal infections and affects immunosuppressed individuals (Maziarz and Perfect, 2016; Velagapudi et al., 2009). *C. gattii*, on the other hand, causes lethal pulmonary infection in apparently immunocompetent individuals although it may also affect immunocompromised patients (Brizendine et al., 2011; Litvintseva et al., 2005; Chaturvedi et al., 2005). Infection occurs when the yeast cells or basidiospores are inhaled resulting in a lung infection followed by a subsequent haematogenous dissemination to the brain; normally determined by both fungal virulence and the host's immune status. Pulmonary cryptococcal infection may present as asymptomatic chest infection (in normal hosts) or acute pneumonia which may rapidly progress to acute respiratory distress syndrome (Maziarz and Perfect, 2016).

*C. neoformans* has a predilection for the brain and invades the different compartments of the brain leading to inflammation and subsequent damage (Klein et al., 2017). The entry of the fungal cells into ventricular compartment leads to ventriculitis and has been associated with the increased intracranial pressure observed in patients with CNS cryptococcosis (Colombo and Rodrigues, 2015). The presence of the fungus in the meningeal compartment is associated with meningitis, while invasion of the parenchyma by the fungal cells is associated with encephalitis (see review by (Colombo and Rodrigues, 2015)). There is considerable evidence available to show that cryptococcal cells can traverse the blood brain barrier (BBB); a natural barrier formed from the endothelium and glia, which restricts the entry of peripheral substances and pathogens circulating in blood from crossing over into the CNS (Chang et al., 2004; Charlier et al., 2005; Santiago-Tirado et al., 2017). Cryptococcal cells can cross the BBB by using transcellular penetration (Chang et al., 2004), Trojan horse transmigration within infected phagocytes (Charlier et al., 2005; Santiago-Tirado et al., 2017) or by disrupting tight junctions to facilitate paracellular transport (Liu et al., 2012). CNS cryptococcosis is typically characterised by headaches, a stiff neck, cranial neuropathies, meningismus (irritation of meninges), fever, and sensitivity to light (Maziarz and Perfect, 2016; Casadevall et al., 2018; Graybill et al., 2000; Makadzange and McHugh, 2014). Patients with chronic neurocryptococcosis may develop psychosis or mania, attention deficits, decreased executive function, loss of vision, and/or loss of hearing (Rothe et al., 2013; Molloy et al., 2021; Klein et al., 2017).

## 4 Pertinent questions on the neuroimmune response to cryptococcal infections

The pathogenesis of cryptococcal meningitis is centred around fungal characteristics and the host-immune response to them. An inadequate inflammatory response is associated with increased mortality due to an increase in fungal burden, while exacerbated inflammatory responses are associated with damage-related morbidity and mortality (Jarvis et al., 2015; Boulware et al., 2010; Vlasova-St Louis et al., 2021b). The innate immune response to peripheral cryptococcosis has been extensively described (see reviews by (Campuzano and Wormley, 2018; Yang et al., 2017)), but huge knowledge gaps still exist in our knowledge of the CNS defence mechanisms against the fungus (Drummond, 2018). The peripheral response to cryptococcus either follows a Th1-type response in which: interferon gamma (IFN-γ) and interleukin (IL) 12 are produced; macrophages are classically activated to release nitric oxide, which is fungicidal; and the pathogen is subsequently cleared by the adaptive immune response (Hoag et al., 1995, 1997; Eisenman et al., 2007). Alternatively, it follows a Th2-type response in which: IL4, IL10 and IL13 are produced; macrophages are alternatively activated and fail to kill the fungus; and there is failure to activate cell-mediated responses for fungal clearance (Eisenman et al., 2007; Jain et al., 2009; Osterholzer et al., 2009; Ellerbroek et al., 2004). Using its multiple virulence factors (capsular polysaccharides, laccase and urease), *C. neoformans* promotes the non-protective Th2 polarisation pattern which favours its persistence and dissemination to the CNS (Eisenman et al., 2007; Jain et al., 2009; Osterholzer et al., 2009; Ellerbroek et al., 2004; Qiu et al., 2012). In mice, the Th polarisation pattern is strain specific. C.B-17 mice have been shown to exhibit resistance to cryptococcal infection by developing a Th1 response while C57BL/6 mice are susceptible and do not develop a Th1 response (Hoag et al., 1997; Hardison et al., 2012). Although the peripheral innate immune system is important for pathogen recognition and initiating pathogen clearance, effective cryptococcal clearance requires recruitment of clusters of differentiation (CD)4^+^ and CD8^+^ cells; which explains why HIV patients with low T-cell counts show a poor inflammatory response and severe disease burden (Bicanic et al., 2009b; Jarvis et al., 2013; Siddiqui et al., 2005; Wozniak et al., 2012).

In the CNS, the events that occur after fungal entry into the parenchyma are less clearly defined. From what is currently available in literature, we know that cryptococcal cells express immunogenic pathogen associated molecular patters (PAMPs), which include: the capsular polysaccharides, glucuronoxylomannan (GXM) and galactoxylomannan (GalXM); mannoproteins, and β-glucans and chitin, which make up the cell wall (Campuzano and Wormley, 2018; Snarr et al., 2017). These PAMPs are recognised by toll-like receptors (TLRs), C-type lectin receptors (CLRs) and NOD like receptors (NLRs), all of which are classified as pathogen recognition receptors (PRRs) (Campuzano and Wormley, 2018; Chen et al., 2017). TLR2 and TLR4, in conjunction with CD14 and CD18, are likely the major PRRs that recognise cryptococcal cells and downstream signalling is dependent on the adaptor molecule, myeloid differentiation factor (MyD)88 (Biondo et al., 2005; Dong and Murphy, 1997; Shoham et al., 2001a; Yauch et al., 2004). Other TLRs reported to contribute to the neuroimmune response to *Cryptococcus* include TLR1/2, TLR3, TLR9 (Nakamura et al., 2008; Redlich et al., 2013) but it seems CLRs, like Dectin 1 and 3, are not required for host defence against *Cryptococcus* (Campuzano and Wormley, 2018; Nakamura et al., 2007; Walsh et al., 2017; Campuzano et al., 2017).

**Fig. 1:**
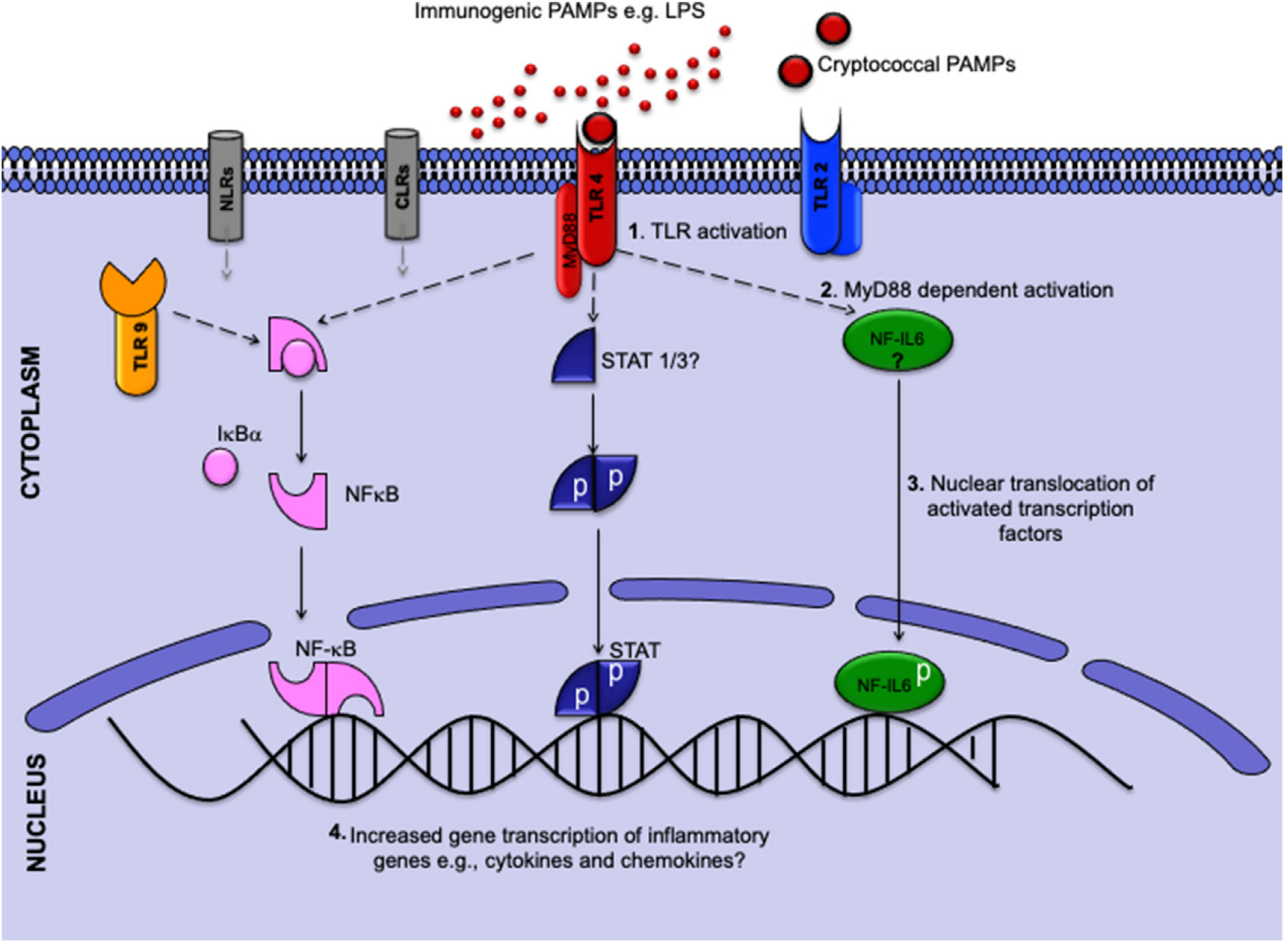
A simplified schematic diagram showing inflammatory signalling pathways activated by toll-like receptor (TLR) agonists and that could subserve the proinflammatory response of neuroimmune cells to *Cryptococcus*. (**1**) Pathogen associated molecular patterns (PAMPs) bind to and activate the TLR. (**2**) Through a myeloid differentiation factor (MyD)88-dependant route (dashed black arrows), there is a downstream activation of inflammatory transcription factors; nuclear factor (NF) for interleukin 6 (IL6), NF-kappa B(NFκB) and signal transducer and activator of transcription (STAT). (**3**) Activated transcription factors translocate to the nucleus where they (**4**) increase the expression of inflammatory genes. Other abbreviations: LPS ​= ​lipopolysaccharide, CLR = C type lectin receptor, NLR, NOD like receptor, IκBα ​= ​nuclear factor of kappa light polypeptide gene enhancer in B-cells inhibitor, alpha. NB: For purposes of clarity, only three classical pathways directly activated by TLRs have been shown in the diagram; pathways involving CLRs and NLRs (grey dashed arrows) have not been included in the illustration.

The molecular signatures of signalling and transcription involved in cryptococcal defences in the brain remain to be fully studied but the activation of TLRs 2 and 4 by their agonists typically leads to the release of proinflammatory cytokines. We have previously shown that zymosan, a fungal cell wall component of *Saccharomyces cerevisiae* and a known TLR2/6 agonist, induces the activation of the inflammatory transcription factors; nuclear factor (NF) for interleukin 6 (IL6), NF kappa B (NFκB) and signal transducer and activator of transcription (STAT)-3, followed by a subsequent expression of genes encoding proinflammatory cytokines (Dangarembizi et al., 2019). Lipopolysaccharide (LPS), an established TLR4 agonist, also activates similar pathways both *in vivo* and *in vitro* (Damm et al., 2011; Rummel et al., 2011; Wuchert et al., 2008). NFκB mediates the release of proinflammatory cytokines by monocytes during cryptococcal infection (Chen et al., 2017). Additionally, recognition of cryptococcal antigens by macrophages is associated with increased STAT-1 transcripts as well as phosphorylation of the STAT-1 protein (Hardison et al., 2012). GXM has been shown to induce NFκB activation in polymorphonuclear blood cells and RAW264.7 ​cells without activating mitogen activated kinase pathways and the release of tumor necrosis factor alpha (TNF-α) (Shoham et al., 2001b). What is unclear is whether the classical inflammatory signalling pathways for TLR4 signalling, which have been summarised in Fig. 1, are also employed by glia in fighting cryptococcal infection in the brain. Pathways with question marks in the figure represent gaps in our knowledge of signalling mechanisms that could be involved in mediating the neuroimmune response to cryptococcal infection. This constitutes a significant gap in our current understanding of the disease.

The question of whether innate immune cells of the brain release proinflammatory or anti-inflammatory cytokines in response to cryptococcal cells has not been satisfactorily answered. Recent reviews on anti-cryptococcal immunity suggest that a proinflammatory response is the natural course of events after TLR activation (Campuzano and Wormley, 2018; Drummond, 2018). The reasoning behind this suggestion may be that a Th1-type activation of peripheral immune cells during pulmonary cryptococcosis is associated with increased production of proinflammatory cytokines such as (IFNγ), IL12 and IL2 which help to stimulate phagocytic clearance of cryptococcal cells by macrophages (Hoag et al., 1995, 1997; Decken et al., 1998; Mukaremera and Nielsen, 2017); a process that is facilitated by intimate crosstalk between innate and adaptive immune cells. In the brain, heat-killed *C. neoformans* injected intracranially has been also shown to induce the expression of proinflammatory cytokines (Lipovsky et al., 1998). On the contrary however, others have shown that live, encapsulated and acapsular *C. neoformans* could not upregulate the expression of proinflammatory cytokines in BV2 microglial cells lines (Barluzzi et al., 1998). Instead this study showed that the presence of *C. neoformans* suppressed LPS-induced TNF-α release by the microglial cells and the inhibitory effects were ascribed to the capsular components GXM and GalXM (Barluzzi et al., 1998). GXM, the major capsular component of *C. neoformans* has also been shown to induce the production of IL8, an anti-inflammatory cytokine in isolated microglial cells (Lipovsky et al., 1998). Other studies also showed that cultured human microglial cells mostly exhibit a fungistatic than fungicidal response to cryptococcal cells in the absence of opsonisation (Lee et al., 1995a, 1995b). Whether innate immune cells of the brain are skewed towards a proinflammatory or anti-inflammatory role when activated by *C. neoformans* is not clearly described, but available evidence suggests that cryptococcus may elicit immunosuppressive effects through its capsular components. Important to note is the fact that even in the periphery, a dichotomous response pattern is observed which is dependent on the virulence factors released by the fungus as much as it depends on the host response. Because it develops and employs virulence factors to facilitate its dissemination and invasion of the brain, it is likely that cryptococcus may suppress the inflammatory responses in the brain in the same manner it suppresses macrophages in the periphery.

## 5 Perspectives for future research: potentially useful experimental models and techniques

### *5.1* *In vivo* models

Studying the neuroimmune response to cryptococcal infection requires one to choose an appropriate model that ensures that the fungus disseminates and successfully colonises the brain to cause disease. Mice, rats and guinea pigs have all been used for studying neurocryptococcosis but most researchers use mice. Infection in rodent models can be induced intravenously, or through the intranasal or intratracheal routes (Coelho et al., 2019; Nielsen et al., 2005; Krockenberger et al., 2010; Park et al., 2018; Riera et al., 1983). Dissemination of the fungus after infection can be visualised using general histological stains such as methenamine silver, periodic acid-Schiff, mucicarmine, or haematoxylin and eosin stains. A recent study developed a model that uses bioluminescence imaging to track the dissemination of a genetically engineered *C. neoformans* strain (KN99α) in mice (Vanherp et al., 2019). The advantage of this novel model is the ability to assess fungal dissemination and disease progression in living rodents using non-invasive imaging techniques such as micro-computed tomography and magnetic resonance imaging.

Genetically modified rodent models are available that could be useful for studying neuroinflammation in cryptococcosis. Using these models, researchers can knock-out genes encoding pathogen receptors, transcription factors, cytokines and chemokines to investigate their roles during the pathogenesis of the disease. Transgenic mice expressing gene-specific reporters can be useful cell-type markers to investigate the role of different cell types in the neuroimmune response to cryptococcal infection. The Cre-(knock-out/knock-in) mouse lines have proven invaluable for visualisation and manipulation of specific glial cell lines. Examples include the popular GFAP-CreERT2 mouse lines for studying astrocytes, and Sall1-CreER, Hexb-Cre and CX3CR1-CreER mouse lines for studying microglia (Eyo et al., 2015; Hu et al., 2020; Kaiser and Feng, 2019; Ruan et al., 2020; Masuda et al., 2020).

Another potentially useful model for studying neuroimmune interactions during cryptococcal infections is the zebrafish. When infected intravenously with *Cryptococcus*, zebrafish develop cryptococcosis similar to murine models; exhibiting latency, peripheral infection and CNS invasion (Davis et al., 2016; Varela et al., 2017). The advantages of the zebrafish as a model for studying neuroimmune responses to cryptococcal infections are: they have optical transparency (allows real-time live imaging) and they have a highly developed immune system with TLRs and signalling systems that are organised in a similar manner to those of rodents and humans (Varela et al., 2017; Novoa and Figueras, 2012; Sullivan and Kim, 2008). Additionally, the whole genome of the zebrafish is available online (http://www.ensembl.org/Danio_rerio/Info/Index) and numerous transgenic zebrafish lines are available with knock-in or knock-out genetic profiles for mechanistic dissection of the role of specific genes or cell types in mediating the neuroimmune response to infection (Kesavan et al., 2018).

### *5.2* *In vitro* models

*In vitro* models offer a potentially powerful platform for delineating neuroimmune mechanisms underlying the pathogenesis of neurocryptococcosis at the cellular and molecular levels. Unlike whole organism models, culture systems allow researchers to perform a close-up analysis of the behaviour, morphology and activation states of specific cell types, and to make direct measurements of releasable factors. Currently, *in vitro* systems that are used or have potential use for neuroinflammation work in cryptococcosis include: dissociated monocultures, in which only one cell type is isolated and maintained in culture, mixed cultures, in which a mixture of different types of dissociated cells are cultured, and organotypic slice cultures, in which slices of the brain or spinal cord are maintained alive in culture (Redlich et al., 2013; Lipovsky et al., 1998; Lee et al., 1994, 1995a; Olave et al., 2017; Czapiga and Colton, 1999; Humpel, 2015; Huuskonen et al., 2005). Monocultures and mixed neuroglia cultures are established models in neuroimmune studies but the organotypic slice culture model is new in the field of neuroinflammation research. Organotypic cultures offer several advantages that could make them useful for studying neuroimmune responses in cryptococcosis: (1) brain tissue slices maintain their three-dimensional cytoarchitecture, connectivity and functional interactions with neighbouring cell types, (2) the cultured tissue maintains a full vascular network with functional endothelial cells, and (3) the living brain tissue allows for controlled manipulations and *in vivo* imaging to show the dynamic changes occurring within tissue in response to a pathogen (Czapiga and Colton, 1999; Humpel, 2015; Huuskonen et al., 2005).

### 5.3 Molecular techniques and next-generation sequencing

Several molecular techniques such as immunohistochemistry, ribonucleic acid (RNA) sequencing (RNA-Seq), flow cytometry and bioassays for measuring releasable factors could all be useful to help decipher the molecular mechanisms underlying CNS cryptococcosis. Using double immunohistochemistry, cytokine bioassays and real-time quantitative polymerase chain reaction, we have previously characterised the inflammatory signalling pathways underlying *Saccharomyces cerevisiae*-induced neuroinflammation in rodents (Dangarembizi et al., 2018). For higher throughput cytokine and chemokine analyses, multiplex assays such as Luminex, may proffer an advantage over standard enzyme linked immunosorbent assays.

Transcriptomic mechanisms underlying the host neuroimmune response to cryptococcosis can be studied using single nuclear RNA sequencing (snRNA-Seq), a next-generation sequencing technique that allows quantitative measurements of RNA in a single cell. SnRNA-Seq offers a high resolution characterisation of the neuroimmune response different cell types, subtypes and activation states. A few studies have described successful characterisation of cell-type specific transcriptomic changes that occur in host cells during fungal infections, including *C. neoformans* (Li et al., 2019; Liu et al., 2014, 2015; Niemiec et al., 2017). A recent prolific study used snRNA-Seq to characterise transcriptomic biomarkers in patients who survived, against those who succumbed to cryptococcal immune reconstitution inflammatory syndrome (Vlasova-St Louis et al., 2021b). Recent advances in RNA-Seq technology now allow for *in situ-*transcriptomic analysis which combines immunofluorescence and RNA-Seq to enable visualisation of the cells from which specific genes are expressed and thus correlate gene expression to morphology, location and cell connections (Avital et al., 2017; Bergenstråhle et al., 2020). Additionally, dual RNA-Seq now potentiates sequencing both host and pathogen to help us understand the events occurring at the host-pathogen axis; the effect of the pathogen on the host and that of the host on the pathogen (Niemiec et al., 2017; Avital et al., 2017; Baddal et al., 2015; Rienksma et al., 2015; Westermann et al., 2017).

## 6 Conclusion

Cryptococcal meningitis is a neglected threat to global brain health and particularly a challenge to African brain health. Although a lot of research is currently being done on understanding the pathogenesis of the disease in the lung, not much focus has gone into understanding the mechanisms underlying its development in the brain. Gaps still exist in our knowledge of the pathogenesis of this fatal infection at cellular and molecular level. The interaction of the fungus with the immune cells of the brain has not been fully characterised. How fungal virulence affects these interactions remains undescribed. And lastly, the significance of the brain's response to the fungus on the development of symptoms has also not been fully described. Considering the urgent need there is to develop cheaper, safer, and more effective drugs to treat the disease, researchers could take advantage of new models and advanced molecular techniques to generate high throughput data that can broaden our understanding of the mechanisms underlying cryptococcosis at the level of the brain.
